# Metabolic Impact on the Hypothalamic Kisspeptin-Kiss1r Signaling Pathway

**DOI:** 10.3389/fendo.2018.00123

**Published:** 2018-03-28

**Authors:** Fazal Wahab, Bibi Atika, Farhad Ullah, Muhammad Shahab, Rüdiger Behr

**Affiliations:** ^1^Platform Degenerative Diseases, German Primate Center, Leibniz Institute for Primate Research, Göttingen, Germany; ^2^Department of Developmental Biology, Faculty of Biology, University of Göttingen, Göttingen, Germany; ^3^Department of Zoology, Islamia College University, Peshawar, Pakistan; ^4^Laboratory of Reproductive Neuroendocrinology, Department of Animal Sciences, Faculty of Biological Sciences, Quiad-i-Azam University, Islamabad, Pakistan; ^5^DZHK (German Center for Cardiovascular Research), Partner Site Göttingen, Göttingen, Germany

**Keywords:** *Kiss1*, kisspeptin, Kiss1r, metabolism, reproduction, metabolic hormones, proopiomelanocortin, AgRP

## Abstract

A large body of data has established the hypothalamic kisspeptin (KP) and its receptor, KISS1R, as major players in the activation of the neuroendocrine reproductive axis at the time of puberty and maintenance of reproductive capacity in the adult. Due to its strategic location, this ligand-receptor pair acts as an integrator of cues from gonadal steroids as well as of circadian and seasonal variation-related information on the reproductive axis. Besides these cues, the activity of the hypothalamic KP signaling is very sensitive to the current metabolic status of the body. In conditions of energy imbalance, either positive or negative, a number of alterations in the hypothalamic KP signaling pathway have been documented in different mammalian models including nonhuman primates and human. Deficiency of metabolic fuels during fasting causes a marked reduction of *Kiss1* gene transcript levels in the hypothalamus and, hence, decreases the output of KP-containing neurons. Food intake or exogenous supply of metabolic cues, such as leptin, reverses metabolic insufficiency-related changes in the hypothalamic KP signaling. Likewise, alterations in Kiss1 expression have also been reported in other situations of energy imbalance like diabetes and obesity. Information related to the body’s current metabolic status reaches to KP neurons both directly as well as indirectly *via* a complex network of other neurons. In this review article, we have provided an updated summary of the available literature on the regulation of the hypothalamic KP-Kiss1r signaling by metabolic cues. In particular, the potential mechanisms of metabolic impact on the hypothalamic KP-Kiss1r signaling, in light of available evidence, are discussed.

## Introduction

Kisspeptin (KP), a hypothalamic neuropeptide, and KISS1R/Kiss1r, the KP receptor, are the main components of an important hypothalamic signaling pathway (1, 2). KP and KISS1R are encoded by *KISS1* and *KISS1R* genes, respectively (3, 4). A large body of data has established an important role for the KP-Kiss1r signaling in the initiation of puberty in both non-primate and primate vertebrates (5–8). Loss of function mutations in human *KISS1* or *KISS1R* genes causes absence of or delayed puberty (8–11), whereas a gain of function mutation in *KISS1R* gene results in precocious puberty (12). Likewise, administration of KP in immature rats elicits an early onset of puberty, whereas KP antagonist infusion leads to a delay in the achievement of pubertal hallmarks (5, 13, 14).

**Figure 1:**
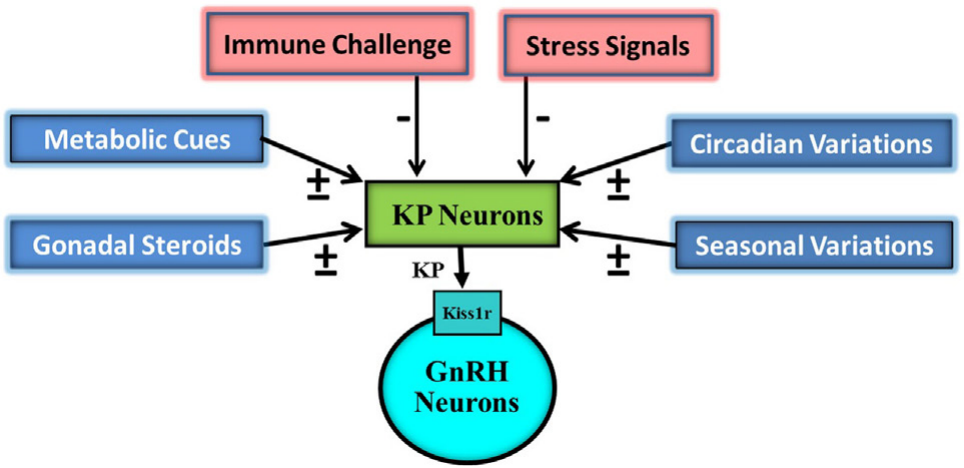
Schematic representation of impact of various external and internal signals on the hypothalamic Kisspeptin (KP) system. KP neurons are targeted by gonadal steroids, metabolic, circadian, seasonal, immune, and stress signals. Some of gonadal steroids, metabolic, circadian, and seasonal signals result in upregulation (+) of KP expressions while others in downregulation. Immune and stress signals cause down regulation (−) of KP expression. KP neurons then on the basis of this information modulate pulsatile discharge of gonadotropin-releasing hormone (GnRH) from GnRH neurons.

Kisspeptin signaling also plays an important role in the maintenance of the reproductive capacity in the adult (1, 15–17). Administration of KP, peripherally as well as centrally, has been reported to markedly increase systemic levels of reproductive hormones both in normal as well as subjects with reproductive insufficiency phenotype (7, 15, 18, 19). Due to their strategic position in the hypothalamus, the KP-containing neurons also act as a conduit for transferring information related to a number of different intrinsic and extrinsic cues to the gonadotropin-releasing hormone (GnRH) neurons (Figure 1). These neurons are involved in circadian and seasonal regulation of reproduction (20, 21). Moreover, this ligand-receptor pair acts as an integrator of the action of gonadal steroids and metabolic cues on the reproductive axis (22–26).

Proper functioning of the hypothalamic KP signaling is very sensitive to the current metabolic status of the body (23, 25) (Table 1). Conditions of energy imbalance, either positive or negative, induce a number of alterations in the hypothalamic KP signaling pathway in different mammalian experimental animal models (22, 26, 27). Deficiency of metabolic fuels during fasting causes a clear reduction of the *Kiss1* gene transcript levels in the hypothalamus and hence decreases the output of KP-containing neurons (5, 28). Food intake or exogenous supply of metabolic cues, such as leptin, overcomes metabolic insufficiency-related changes in the hypothalamic KP signaling (29, 30). Likewise, alterations in *Kiss1* expression have also been reported in other situations of energy imbalance like diabetes and obesity (30, 31). All these findings indicate a high sensitivity of KP signaling to alterations in the body’s energy homeostasis. In this review, we summarize and discuss the presently available pieces of evidence indicating an impact of metabolic status-related cues on the hypothalamic KP-Kiss1r signaling in conditions of energy imbalance. We also discuss potential mechanisms of the transmission of the metabolic information on the hypothalamic KP system and ultimately reproduction.

**Table 1:** Effect of different metabolic hormones and neuropeptides on the hypothalamic Kisspeptin (KP) system under different experimental setup in rodents and primates.

Hormone/neuropeptide	Effect on KP	Experimental setup	Experimental model	Reference
Adiponectin	↓	*In vivo* and *in vitro*	Mouse	(58)
Leptin	↑	*In vivo*	Mouse and rat	(30, 31, 33)
Ghrelin	↓	*In vivo*	Mouse and rat	(80, 81)
Insulin	↑ =	*In vivo* and *in vitro*	Mouse and sheep	(30, 89)
Melanocortin	↑	*In vivo*	Mouse	(126)
Glucagon-like peptide 1	↑ =	*In vitro* and *in vivo*	Mouse	(128)

*Increase (↑), Decrease (↓), no effect (=)*.

## Sensitivity of the Hypothalamic KP-Kiss1r Signaling Pathway to Metabolic Alterations in Conditions of Altered Energy Homeostasis

**Figure 2:**
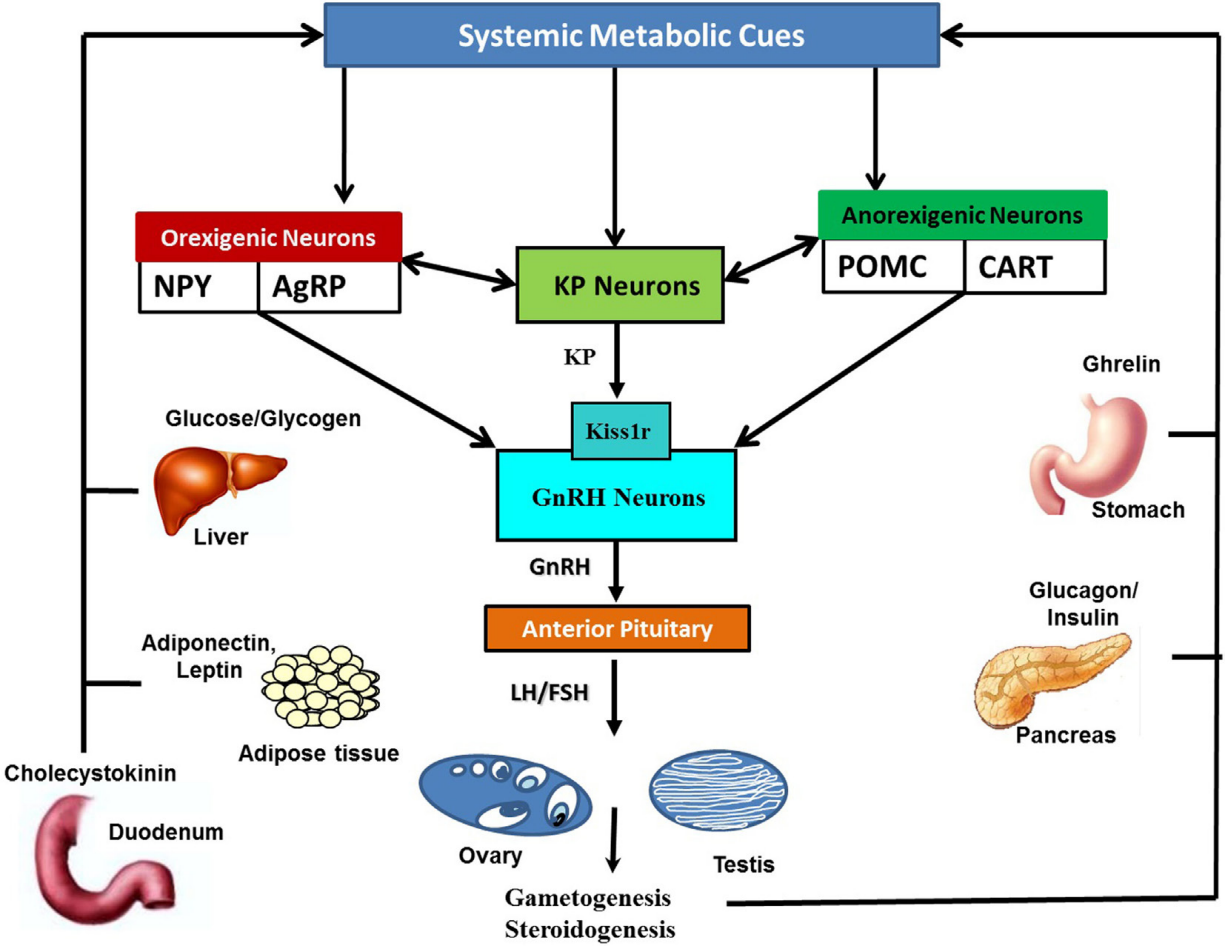
Schematic representation of the interaction of systemic metabolic cues with Kisspeptin (KP), orexigenic, and anorexigenic neurons: metabolic cues are secreted by metabolic organs in responses to alterations in metabolic status. Metabolic cues include insulin and glucagon from pancreas, leptin, adiponectin and leptin from adipose tissues, ghrelin from stomach, glucose, fatty acid, cholecystokinin, glucocorticoids, and thyroid hormones, among many others. Alterations in metabolic cues, either directly or indirectly *via* anorexigenic and orexigenic neurons, modulate KP neuronal activities. KP neurons in turn transfer this information to the HPG axis *via* gonadotropin-releasing hormone (GnRH) neuronal network. Likewise, orexigenic and anorexigenic neurons can also directly convey current metabolic status related information to GnRH neurons.

The hypothalamic KP-Kiss1r system is highly sensitive to alterations in the metabolic cues levels in the systemic circulation. All sorts of metabolic perturbances exert negative impact on the *Kiss1* expressing neurons (Figure 2) (22, 23, 26, 27, 32). It is well established that reduction of metabolic fuels in food-deprived conditions causes a decrease in *Kiss1* transcript levels in the arcuate nucleus (ARC) (5, 28). In some conditions of energy imbalance, such as diabetes and obesity, very high energy reserves are present in the body, but due to the body’s inability to properly utilize them, an attenuation of *Kiss1* mRNA expression was observed (30, 31, 33).

Both direct and indirect evidence suggests that deficiency in metabolic fuels severely affects the KP neuronal network in the hypothalamus. Short-term fasting-associated metabolic cues alterations lead to a marked reduction of hypothalamic *Kiss1* expression in prepubertal as well as adult rats (5, 29, 34–36). Castellano et al. (5) carried out a first comprehensive analysis of the short-term fasting impact on the hypothalamic Kiss1 system in prepubertal rats. In fasted rats, delayed puberty, as monitored by vaginal opening, was associated with a reduction of whole hypothalamic *Kiss1* gene transcript levels. However, *Kiss1r* mRNA expression was increased in these rats as compared to normally fed control animals. A possible explanation, as provided by authors (5), for this opposite change in Kiss1 and Kiss1r is that a major reduction in ligand (KP encoding gene) expression might cause a compensatory increase in the expression of its receptor gene, leading to a situation of sensitization to the effects of KP. Importantly, exogenous administration of KP not only rescues the suppression of the reproductive axis in these rats but also overcomes the negative energy balance-induced pubertal delay (5). This finding indicates that a proper reserve of energy is critical for the achievement of reproductive capacity at the time of puberty. The energy reserve related cues, in turn, communicate with the neuroendocrine center for the regulation of reproduction through the hypothalamic neural circuitry of KP neurons (37). Subsequent studies analyzed the impact of food restriction on the distinct hypothalamic KP neuron subpopulations in ARC and anteroventral periventricular nucleus (AVPV). In adult ovariectomized female rats, fasting decreased AVPV *Kiss1* mRNA levels, but not *Kiss1* mRNA expression in the ARC (34). In the intact adult female rats, food deprivation resulted in a prolongation of the reproductive cycle *via* a reduction in ARC *Kiss1* mRNA expression (34). However, these researchers did not observe any changes in AVPV *Kiss1* mRNA expression. Likewise, chronic food deprivation in pubertal female rats diminished expression of *Kiss1* in ARC, but not in the AVPV (38). In mice and rhesus macaques, in contrast to rats, the hypothalamic transcript levels of both *Kiss1* and *Kiss1r* are reduced by a 48-h fast (28, 30).

In addition to the aforementioned expression data, KP administration data also indirectly pinpointed a high sensitivity of the hypothalamic KP system to fasting-induced negative energy balance (5, 39). Administration of exogenous KP has been documented to overcome the negative energy balance-induced suppression of the reproductive axis, further advocating the idea that the endogenous KP system is negatively affected by fasting (5, 39).

Besides the condition of fasting, experimental data from other paradigms of energy imbalance such as diabetes, obesity, and lactation also indicate an impact of metabolic perturbations on the KP neurons output (30, 31, 40). The hypothalamic expression of *Kiss1* gene is significantly reduced not only in the rat model of diabetes but also in obesity rodent models (30, 31, 40). In both congenital leptin deficiency and high-fat-diet-induced models of obesity, Kiss1-expressing neurons output is greatly reduced (33, 40). Likewise, a reduction in *Kiss1* expression has also been reported in lactating female rats (41, 42). Moreover, exogenous administration of KP has been noted to rescue the energy imbalance impact on the reproductive axis (41, 42).

Taken together, the evidence summarized above strongly suggests a very high sensitivity of KP-containing neurons to metabolic alterations in the body.

## Mechanism of Metabolic Impact on the Hypothalamic KP System

The exact mechanism by which changes in metabolic cues alter the hypothalamic KP system is still not fully clear. Available data suggests both direct and indirect mechanisms. Hypothalamic KPergic neurons can most likely sense metabolic cues directly because receptors for a number of peripheral metabolic hormones have been shown to be expressed by these neurons (22, 23, 25, 26, 32). Indirect sensing of metabolic status-related information is also possible because KP neurons receive information from various neuronal networks by direct cell-cell-communication, and neurons capable of sensing systemic metabolic cues are part of these networks (23, 25, 32, 43, 44). In this section, we summarize available data on both direct and indirect impact of metabolic cues on the hypothalamic KPergic neurons.

### Direct Impact of Peripheral Metabolic Factors on KP Secreting Neurons in the Hypothalamus

#### Adiponectin

Adiponectin, a white adipocyte-secreted adipocytokine, was first documented in 1995 independently by various groups (45–48). It is a 244 amino acid protein hormone encoded by the *APMI* gene. It is secreted in very large amount into the systemic circulation. It has been noted to be about 0.01–0.05% of the total systemically circulating proteins (45–49). Systemic concentration of adiponectin is ranged from 3 to 30 µg/mL (45). Adiponectin levels are sexually dimorphic as its concentration is higher in females than in males (45). In various metabolic disorders, such as obesity and diabetes, a marked reduction in plasma adiponectin levels has been reported (49, 50). Nevertheless, its levels are markedly elevated during fasting and are positively associated with severe weight reduction although in these situations the body has a greatly reduced adipose tissue mass (51, 52). This elevation in plasma adiponectin levels during food restriction condition is caused by adipose tissue in bone marrow. In contrast to other parts of the body, a prominent increase in the mass of adipose tissue in bone marrow has been noted in food restriction conditions (53).

Adiponectin exerts its biological action *via* two 7-transmembrane receptors, AdipoR1 and AdipoR2 (45, 51), which are structurally as well as functionally different from 7-transmembrane G protein-coupled receptors. These receptors constitute a subgroup of 7-transmembrane receptors together with 11 progestin AdipoQ receptors (45). Besides peripheral organs, studies have demonstrated expression of both AdipoR1 and AdipoR2 in various brain regions, including the hypothalamus, although evidence for the transport of adiponectin across the blood-brain barrier is still lacking (54–56).

Binding of adiponectin to its receptor leads to the activation of 5’ AMP-activated protein kinase (AMPK). The activated AMPK acts to regulate energy homeostasis of the cell *via* fatty acid oxidation and stimulation of glucose uptake (45, 49, 51). Moreover, adiponectin has been shown to modulate the release of reproductive hormones. Adiponectin inhibits LH, GnRH-stimulated LH, and GnRH secretion while no impact on follicle-stimulating hormone (FSH) secretion was noted (54, 56). Recently, Wen et al. (57). analyzed the adiponectin effect on hypothalamic *Kiss1* mRNA expression in GT1-7 cells, which are immortalized mouse hypothalamic neuronal cells, and *in vivo* in rats. They showed that adiponectin, as well as a synthetic activator of AMPK, greatly reduced transcription of *Kiss1* mRNA while inhibition of AMPK caused an increase in expression of *Kiss1* mRNA in both *in vitro* and *in vivo* studies. Taken together, these findings suggest a negative impact of adiponectin on the activities of KP-containing neurons. The negative impact of adiponectin on Kiss1 expression suggests that it might be involved in short-term fasting induced suppression of the reproductive axis. In fasting condition, an increase in systemic levels of adiponectin has been reported.

#### Leptin

Leptin is another important adipokine of white adipose tissue. In contrast to systemic adiponectin levels, leptin levels in the bloodstream are directly related to the body mass of adipose tissues. Leptin plays a vital role in the maintenance of energy balance in the body (58–60). One of the key functions of leptin is to communicate information on the body’s current metabolic status to brain centers for energy homeostasis (61, 62). Systemic concentrations of leptin are reduced in food restriction conditions while food intake augments leptin concentrations (63). Available experimental data show that leptin is an important regulator of the metabolic deficiency/sufficiency-induced alterations in the neuroendocrine axes. Thereby, it also affects reproductive functions (37, 60, 64).

Besides peripheral reproductive organs, expression of the leptin receptor (LepR) has also been noted in several central neuronal networks in the hypothalamus, including KP-secreting neurons (33, 37). In situations of energy imbalance, low levels of leptin cause a clear reduction in *Kiss1* transcripts levels in the hypothalamus (28, 30, 31, 33, 40) while the elevation of systemic leptin concentrations *via* exogenous administration greatly ameliorates expression of *Kiss1* transcripts levels (30, 33). Similarly, ablation of leptin in *ob/ob* mice and hypoleptinemia in experimental diabetic rats diminish *Kiss1* mRNA expression while leptin infusion in both, ob/ob mice and in the rat model augments *Kiss1* transcript levels (30, 31, 33). Leptin can also indirectly change activities of KP-secreting neurons because many studies have reported the expression of LepR in numerous discrete regions of the hypothalamus (58). Important neuronal populations that express LepR include the GABAergic, neuropeptide Y (NPY), proopiomelanocortin (POMC), and agouti-related peptide (AgRP) populations (44, 58, 65, 66). These neurons are known to communicate with KP neurons (43, 44, 67). The indirect impact of leptin on KP neurons is supported by the evidence that exogenous leptin injection was unable to induce signal transducer and activator of transcription-3 (STAT3), a leptin action mediating intracellular signaling pathway, expression in KP neurons (65).

However, Donato et al. (68) have recently shown that hypothalamic KP neuronal LepR deletion did not change LH secretion. Likewise, re-expression of LepR on KP cells in LepR null mice also did not improve hypogonadotropic hypogonadism phenotype in these mice (69). These observations, together with above mentioned findings (28, 30, 31, 33, 40) of a pivotal role of leptin in KP secretion, suggest a potential developmental compensation or an indirect effect of leptin in modulating KP secretion in mice. Nevertheless, more studies are required in other species to further clarify the link between leptin and KP.

#### Ghrelin

Ghrelin, an orexigenic peptide hormone of the upper gastrointestinal track, is a ligand of growth hormone secretagogue receptor (GHSR), which is also a member of the seven transmembrane receptor family (70–72). Ghrelin has been implicated in the short-term regulation of food intake. The systemic concentrations of ghrelin increase at the preprandial time, whereas they decrease postprandially (70, 72, 73). In food restriction conditions, increased ghrelin levels in the circulation are associated with a decrease in reproductive hormones (74). Exogenous ghrelin administration rapidly induces food intake and inhibits the reproductive axis (70, 72, 74, 75). Besides short-term actions on food intake, ghrelin is also involved in the regulation of long-term body weight. Chronic administration of ghrelin increases the body weight through a number of mechanisms, including continuous stimulation of food intake, alterations in energy expenditure, and induction of adiposity (75). In mice, congenital loss of ghrelin or of the *GHSR* gene causes resistance to high-fat-diet-induced adiposity and weight gain (76, 77). Likewise, ablation of both ligand and receptor resulted in reduced body weight of mice, high energy expenditure, and increased motor activity on a standard chow without exposure to a high-fat diet (78). All in all, the available data pinpoint an important role of ghrelin in monitoring and transferring metabolic information to the brain centers implicated in the regulation of reproduction and intake of food intake.

Ghrelin acts centrally in the brain *via* GHSR in the hypothalamus to stimulate food intake and to alter reproduction (72, 75). Expression of GHSRs has been observed on a subset of *Kiss1*-expressing neurons. In 2009, Forbes et al. (79) reported a reduction in the hypothalamic transcript levels of *Kiss1* in response to an increase in circulating ghrelin levels either due to food deprivation or exogenous injection of ghrelin. Besides this direct action of ghrelin on the hypothalamic *Kiss1* gene expression, an indirect action *via* interneurons like the AgRP/NPY neurons (75), which will be discussed below, is also possible.

An important role of estradiol has been reported in the modulation of KP neuronal response to ghrelin by Frazao et al. (80). These researchers found that elevated levels of estradiol augment transcript levels of GHSR in the hypothalamic ARC. Moreover, an increase in the number of KPergic neurons responding to ghrelin was noted (80). Very recently, it has been reported that an increase in ghrelin levels during the short-term fasting condition leads to a stimulatory effect of central KP on growth hormone secretion. This effect has not been observed in normal fed condition. Moreover, a ghrelin receptor antagonist or a block of increase in its systemic levels abolishes this effect of KP on growth hormone secretion. On the basis of these findings, it has been proposed that central KP neuronal networks might transfer reproductive and metabolic status related cues onto the somatotropic axis thus causing a change in the release of growth hormone (81).

#### Insulin

Insulin, a metabolic hormone secreted by the pancreatic β cells, is involved in metabolic regulation of reproduction through actions on both central and peripheral components of the reproductive axis (82, 83). Central injections of insulin cause a dose-dependent attenuation in feeding and body weight (84). Ablation of insulin receptor (IR) from neurons results in hypogonadotropic hypogonadism in mice *via* central hypothalamic mechanism (85). Moreover, central injection of insulin has been reported to reinstate normal LH secretion in an experimental rat model of diabetes (86).

Besides many other neurons, expression of IRs has been noted on the ARC KP cells (87). It has been found that the specific deletion of the IR gene from KP neurons delayed the onset of puberty in mice but reproductive capacity was normal in adulthood (87). Therefore, these observations indicate that insulin signaling in KP neurons is important for the normal pubertal awakening of the reproductive axis but not an absolutely critical signal for the achievement of ultimate pubertal hallmarks. Additionally, reproductive ability, feeding, glucose regulation, distribution of fat, and body weight were normal in adult mutants. Of note, administration of insulin in the late follicular ovarian phase significantly stimulated expression of the c-fos protein in sheep ARC KP neurons (88), although it is not clear whether this effect is direct or indirect.

Some indirect evidence supports a possible role of insulin in altering the activity of hypothalamic KP-secreting neurons. In rats, experimental chronic diabetes has been noted to cause a marked reduction of *Kiss1* transcript levels in the hypothalamus (31, 89). Likewise, during short-term fasting, which is characterized by reduced levels of insulin, a reduction in *Kiss1* expression was reported (28, 90). However, exogenous injections of insulin did not reverse the decreased *Kiss1* gene expression, which was induced by fasting- and diabetes-associated metabolic perturbations (30, 31). Additionally, *in vitro* data did not show any effect of insulin on KP expression in the mouse hypothalamic cell line N6 (30). Of note, leptin and NPY applications have stimulated *Kiss1* expression in this cell line.

### Indirect Impact of Metabolic Cues on the Hypothalamic KPergic Neurons

Besides the *per se* impact of metabolic cues on the hypothalamic KPergic neurons, a number of other hypothalamic neuronal networks are also sensitive to metabolic status-related cues. The major hypothalamic neuronal systems, which express the LepR, the GHSR, and the IR, include gamma-aminobutyric acid (GABA), glutamate, NPY/AgRP, and POMC/CART neurons. Many of these neurons, in turn, can alter activities of the hypothalamic KPergic neurons either directly or indirectly.

#### Glutamate and GABA Expressing Neurons’ Input to KP Secreting Neurons in the Hypothalamus

Glutamate and GABA neurons are playing important roles in the regulation of reproduction (91). These neurons have been documented to contain receptors for metabolic hormones, and their activities are modulated by metabolic cues (44, 67, 92–94). In a recent study, we checked changes in the hypothalamic glutamate and GABA systems in fed and 48 hours fasted monkeys *via* checking transcripts levels of *Kiss1, Kiss1r, NR1* (*N*-methyl-d-aspartate receptor subunit 1) and *GAD67* (glutamic acid decarboxylase67) in the mediobasal hypothalamus (MBH) and pre-optic area (POA) of the adult male rhesus macaque (*Macaca mulatta*) (95). The expression of *Kiss1, Kiss1r*, and *NR1* mRNA was greatly decreased in fasted macaques as compared to *ad libitum* fed monkeys. A noteworthy reduction was also noted in the expression of KP and the interactions of NR1 with KPergic neurons in the hypothalamus of fasted monkeys. Taken together, these observations indicate that a reduction in inputs of glutamate-containing neurons to KPergic neurons may be responsible for the reduction in the hypothalamic KP signaling in the fasted monkey. However, no obvious change in expression of *GAD67* mRNA between fed and fasted monkey was observed, suggesting that the fasting-induced reduction in the hypothalamic KP signaling is not mediated through GABAergic neurons (95).

#### RFamide-Related Peptide-3 Expressing Neurons Input to KP-Secreting Neurons in the Hypothalamus

The hypothalamic gonadotropin-inhibitory hormone and its mammalian ortholog RFamide-related peptide-3 (RFRP-3) neurons have been implicated as the potent inhibitors of reproduction in a number of vertebrate species (26, 96–98). RFRP-3 binds to a G protein-coupled receptor namely GPR147. GPR147 is expressed in different regions of the hypothalamus including a subset of the hypothalamic KPergic neurons in the ARC (99). Moreover, a direct contact between GnIH fibers and about 35% of ARC KPergic neurons was also noted.

Different studies in animal models and human subjects analyzed of RFRP-3 effect on KP stimulation of GnRH (99, 100). In human subjects, although RFRP-3 exerts an inhibitory effect on LH secretion in postmenopausal women, no noteworthy effect of RFRP-3 was observed on KP-stimulated LH secretion in men during concomitant KP and RFRP-3 administration (100). In mouse hypothalamic explant culture, research from Tsutsui’s group showed that RFRP3 significantly reduced KP-induced GnRH release (99). Of note, no effect of RFRP-3 on KP-induced GnRH release was noted in the mouse hypothalamic GT1-7 cells (101).

Leon et al. performed an analysis of the GPR147 ablation on the hypothalamic *Kiss1* mRNA expression (102). They reported that GPR147 null mice showed normal pubertal awakening of the reproductive axis. Of note, an increase in expression of *Kiss1* mRNA was noted in the hypothalamic ARC of the adult GPR147 null male mice. Additionally, an increase in systemic levels of FSH and response of LH to GnRH stimulation was observed in GPR147 null mice. However, ablation of GPR147 did not rescue hypogonadotropic hypogonadism in *Kiss1r*-ablated mice. More importantly, in the GPR147 null mouse energy imbalance conditions induced a lesser degree of disruption in the secretion of LH (102). These findings indicate that a lack of RFRP3 signaling may partly prevent metabolic perturbation induced inhibition of the reproductive axis. However, expression of *Kiss1* mRNA was not checked in GPR147 ablated mice in these conditions of metabolic perturbations. Therefore, it will be important to check *Kiss1* expression in GPR147 null mice in situations of metabolic insufficiency in order to know whether RFRP-3 signaling mediates nutritional challenge induced suppression of the reproductive axis.

#### Orexigenic Neuronal Input to the KP Secreting Neurons in Hypothalamus

Hypothalamic orexigenic neurons include NPY and AgRP neurons among others (103–108). AgRP is utterly secreted by a specific neuronal population in the ARC, which also co-expresses NPY. These neurons are playing a crucial role in feeding. They stimulate feeding when they are activated by metabolic deficiency-associated signals (105, 109).

These neurons express receptors for several key metabolic hormones like leptin, insulin, and ghrelin (103, 107, 109). Hence, AgRP/NPY neurons are direct targets of leptin action (109, 110) Exogenous injection of leptin induces a mark activation of STAT3, a prominent leptin action mediating intracellular signaling pathway, in AgRP/NPY neurons (109–111). Insulin has been noted to inhibit the electrophysiological properties of NPY/AgRP neurons. Insulin causes inhibition of NPY/AgRP neurons through activation of ATP-sensitive K^+^ channels (112). However, ablation of IR from AgRP/NPY neurons does not induce prominent alterations of the reproductive axis, while deletion of both, IR and LepR, adversely affected the reproductive axis (113, 114). Ghrelin stimulates the activity of ARC AgRP/NPY neurons (75, 109, 115) *via* activation of GHSR present on these neurons (109, 116). More importantly, it has been reported that ghrelin’s orexigenic effects are lost in *Agrp* and *Npy* knockout mice, suggesting that intact NPY and AgRP neurons are essential for orexigenic effects of ghrelin (115).

Although a large body of data established the perception of metabolic cues by the AgRP/NPY neurons, there is only very limited information on possible routes *via* which the effects are transmitted to KP neurons in the ARC. In the ovine brain, Backholer et al. (43) observed the occurrence of reciprocal transsynaptic neural connections between the hypothalamic NPY-containing cells and the perikarya of the KP-expressing neurons. This anatomical evidence indicates that NPY-containing neurons can affect the output of KP neurons. More importantly, a normal NPY neuronal circuitry is essential for proper functioning of hypothalamic KPergic neurons, as mice with NPY deficiency have defective hypothalamic KP expression (30).

Recently, Foradori et al. provided more comprehensive evidence for cross-talk between KP and NPY neurons (81), especially from KP to NPY neurons in presence of the fasting-induced alteration in metabolic cues. KP administration in fasted ewe has been noted to cause a significant increase in growth hormone level *via* stimulation of NPY neurons and growth hormone releasing-hormone in ARC and an inhibition of somatostatin neurons in the periventricular nucleus.

#### Anorexigenic Neuronal Input to KP-Containing Neurons in the ARC

The hypothalamic ARC POMC (POMC)/CART (cocaine- and amphetamine-regulated transcript) neurons have been implicated as a pivotal central controller of metabolic homeostasis (109, 117, 118). These neurons have been described to constitute a major part of the hypothalamic satiety center. The anorexigenic role of POMC neurons is pinpointed by the evidence that ablation of the *Pomc* gene results in a state of severe hyperphagia, which ultimately leads to an enormous amount of weight gain (119). Moreover, food deprivation reduces mRNA levels of *Pomc* in the hypothalamic ARC, whereas transcript levels of hypothalamic *Pomc* are augmented in overfed rats (120). Similarly, *CART* mRNA expression is also at the nadir in fasting, while food intake restores ARC *CART* mRNA expression (121).

The possible metabolic cues that may be sensed by POMC neurons include leptin and insulin. Presence of both LepR and IR has been noted on POMC neurons (109, 117, 122–124). Recently, researchers documented *via* the whole-cell recording that both leptin and insulin excite POMC neurons and nearby KP cells *via* stimulation of TRPC5 (short transient receptor potential channel 5) channels (112), which are abundantly present in these hypothalamic neurons. Moreover, central administration of exogenous insulin greatly suppressed feeding and enhanced expression of the c-fos protein in ARC POMC neurons (112).

Indeed, POMC neurons are strategically located in the hypothalamus. Thereby, they can integrate the information provided by many different metabolic cues and can link these to the KP neurons. A direct action of POMC neurons is supported by the presence of reciprocal connections between POMC and KP neurons in the hypothalamus (43, 125). POMC neuronal projections were observed in close apposition with a number of other neurons which cross-talk with KP neurons. This also suggests an indirect connection.

Very recently, Tena-Sempere’s group uncovered a melanocortin-KP-GnRH regulatory pathway (126). This pathway was reported to be involved in transmitting leptin actions and plays an important role in regulating the onset of puberty. Of important note, KP neurons were noted to play a vital role in relaying the stimulatory effects of melanocortin signaling onto the reproductive centers (126). In this regard, they reported the existence of a close contact between α-MSH fibers and KP-containing neuronal cell bodies in the ARC of pubertal female rats while the chronic block of the melanocortin receptor, MC3/4R, results in a significant reduction of *Kiss1* transcript levels. Moreover, the LH responses to the MT-II melanocortin agonist, which stimulates LH release, greatly reduced in Gpr54-ablated mice and also in DREADD-induced inhibition of ARC Kiss1 neurons. Altogether, these findings suggest central role KP in mediating impact of POMC neurons on to GnRH neurons during development and metabolic cues related changes.

Very recently, True et al. have reported several differences in coexpression patterns of various hypothalamic neuropeptides in female nonhuman primates as compared to rodents (127). They did not observe coexpression of CART with POMC but instead with NPY. They also noted co-expression of the CART in a subpopulation of KP cells. These CARP + KP neurons were noted to show close appositions with GnRH neurons. In contrast, the single-labeled KP and CART fibers were in synaptic contacts with GnRH neurons.

Heppner et al. (128) reported that KP neurons in the hypothalamic ARC receive synaptic input from glucagon-like peptide 1 (GLP-1), which is an anorexigenic neuropeptide. Moreover, KP neurons also express Glp1r mRNA. More importantly, they noted an increase in KP neurons action potential firing after application of the GLP-1R agonist. GLP-1R agonist also results in a direct membrane depolarization of ARC KP cells. However, central infusions of the GLP-1R antagonist, exendin (9–39), did not exert any effect on expression of ARC Kiss1mRNA or plasma LH in the normal fed mice (128).

## Conclusion and Future Recommendations

In summary, emerging and increasing evidence indicates that metabolic cues exert a profound impact on the hypothalamic *Kiss1*-expressing neurons, both directly and indirectly. The direct sensing of metabolic cues is indicated by the presence of metabolic hormone receptors on *Kiss1*-expressing neurons while indirect sensing of metabolic information is suggested by cross-talk of these neurons with other hypothalamic neuronal populations which also respond to metabolic cues.

Most of the current evidence for the metabolic regulation of the hypothalamic KP system is provided by non-primate studies. Therefore, in the future, further studies in nonhuman primates are required to get more insight into the mechanism by which various peripheral metabolic cues (leptin, adiponectin, testosterone, estrogen, cortisol/corticosterone, ghrelin, insulin, glucagon, thyroid hormones, etc.) exert effects on their central neuronal targets (KPergic, AgRP/NPY, POMC/CART, GABA, corticotropin-releasing hormone, etc.). Indeed, deeper understanding of the metabolic impact on the hypothalamic KP signaling in animal models phylogenetically closer to humans and therefore with high clinical significance will more likely put Kiss1-Kiss1r signaling in the focus as a potential drug target. This may include improvement and management of reproductive functions as well as treatment of disorders of energy balance. Notably, an important role of KP has been shown in the restoration of the reproductive axis after its quiescence in metabolic disorders such as diabetes and hypothalamic amenorrhea (129, 130).

In 2014, Tolson et al. (131) have shown that ablation of KP signaling leads to a reduction in the body’s metabolic activities. They also noted that a lack of the KP system leads to glucose intolerance and obesity (131). However, it is not known whether KP exerts an impact on metabolic activities peripherally or centrally or both. Therefore, it will be interesting to check the impact of various organ-specific knockdowns of KP signaling on metabolism. Very recently, De Bond et al. (132) compared the expression of different metabolically important genes, such as *Npy, Pomc, lepr, Ghsr* (ghrelin receptor), *Mc3r* (melanocortin receptors 3), and *Mc4r* (melanocortin receptors 4). Unexpectedly, they observed no clear alterations in gonadectomized kiss1r-ablated mice compared to intact controls. These findings indicate that the etiology of obesity in the lack of KP-Kiss1r signaling may show an impairment in metabolic cues peripherally instead of central metabolic impairments (132).

## Author Contributions

FW and BA have written first draft of this review article and have drawn figures. FU, MS, and RB have edited and revised the review article. All authors have read and approved the final version of the manuscript.

## Conflict of Interest Statement

Authors declare that there is no conflict of interest that could be perceived as prejudicing the impartiality of the research reported.

## Abbreviations

KP, kisspeptin; GnRH, gonadotropin-releasing hormone; ARC, arcuate nucleus; AVPV, anteroventral periventricular nucleus; NPY, neuropeptide Y; AgRP, agouti-related protein; POMC, proopiomelanocortin; CART, cocaine- and amphetamine-related transcript; LepR, leptin receptor; IR, insulin receptor; GHSR, growth hormone secretagogue receptor.
